# Tandem Mass Tag-Based Quantitative Proteomic Analysis of Chicken Bursa of Fabricius Infected With Reticuloendotheliosis Virus

**DOI:** 10.3389/fvets.2021.666512

**Published:** 2021-05-25

**Authors:** Dahan Yang, Xiaoping Lv, Shujun Zhang, Shimin Zheng

**Affiliations:** ^1^College of Veterinary Medicine, Northeast Agricultural University, Harbin, China; ^2^Heilongjiang Key Laboratory of Laboratory Animals and Comparative Medicine, Harbin, China; ^3^College of Veterinary Medicine, China Agricultural University, Beijing, China

**Keywords:** reticuloendotheliosis virus, bursa of fabricius, quantitative proteomic, differentially expressed protein, histopathological and ultrastructural change

## Abstract

Reticuloendotheliosis virus (REV) is a type C avian retrovirus that causes immunosuppression, dwarf syndrome, and lymphoma in infected hosts. In this study, we used tandem mass tag (TMT) labeling and liquid chromatography–tandem mass spectrometry (LC-MS/MS) to characterize protein alterations in chicken bursa of Fabricius, before and after REV infection at 7, 14, 21, and 28 days. Our data showed that 1,127, 999, 910, and 1,138 differentially expressed proteins were significantly altered at 7, 14, 21, and 28 days after REV infection, respectively. Morphological analysis showed that REV infection reduced in cortical lymphocytes, bursal follicle atrophy, and nuclear damage. Bioinformatics analysis indicated these proteins were mainly involved with immune responses, energy metabolism, cellular processes, biological regulation, metabolic processes, response to stimuli, and multicellular organismal process. Kyoto Encyclopedia of Genes and Genomes (KEGG) pathway cluster analysis showed that post-infection, proteins were enriched in the cell cycle, Wnt signaling, antigen processing and presentation, cytokine receptor interaction, adenosine 3′,5′-cyclic monophosphate signaling pathway, and NF-κB signaling. In addition, we observed that peroxiredoxin 4 (PRDX4), peroxiredoxin 6 (PRDX6), glutathione peroxidase 3 (GPX3), catalase (CAT), and peroxidasin (PXDN) were involved in oxidative stress. Some heat shock protein (HSP) family members such as HSPH1, DNAJA4, HSPA8, and HSPA4L also changed significantly after REV infection. These findings help clarify interactions between REV and the host and provides mechanistic insights on REV-induced host immunosuppression.

## Introduction

In poultry, reticuloendotheliosis virus (REV) infection leads to immunosuppression, acute reticuloma, short stature syndrome, and lymphoid and other chronic tumorous proliferative diseases (1). REV prevalence is primarily combined with other immunosuppressive viruses such as Marek's disease virus (MDV) and subgroup J avian leukosis virus, which complicate disease diagnosis and prevention (2, 3). Also, REV-mediated immunosuppression increases the risk of host infection with other pathogenic microorganisms and decreases immune responses to vaccines (4, 5). The bursa of Fabricius is the main center for B cell development, maturation and differentiation, and REV infection. Viral infection also affects humoral immune responses. Therefore, we studied the proteomics of the bursa of Fabricius after REV infection to understand REV pathogenic mechanisms and different immune responses against REV (6).

During infection, host cell protein abundance undergoes tremendous change, which potentially reveals the viral impact on host cells and organs and also provides useful information on the pathogenesis of viral infection (7–10). In recent years, proteomics has become an important method for studying host cell responses to viral infection and provides new research directions for studying viral pathogenic mechanisms (11, 12). In a transcriptomic study by Yu et al., gene changes in the bursa of Fabricius of REV-infected specific-pathogen-free (SPF) chickens were analyzed (13). Xue et al. studied proteomic changes in chicken spleen infected with REV and identified 28 differentially expressed proteins (14). However, because mRNA expression is not completely consistent with protein expression, data from transcriptomic and proteomic studies are often inconsistent (15, 16). To facilitate such studies, *in vitro* tandem mass tag (TMT) peptide labeling, combined with liquid chromatography–tandem mass spectrometry (LC-MS/MS) quantitative proteomics, has improved protein analysis and identification (17). The bursa of Fabricius is an important immune organ in poultry and plays an important role in host–virus infections. Infectious bursal disease virus (IBDV) leads to host inflammatory cytokine imbalance, reduced immune responses, and immune injury in SPF chickens (18, 19). However, proteomic changes in the bursa of Fabricius after REV infection in SPF chickens, using TMT-labeled quantitative proteomics, have not been reported.

In this study, we used this approach to investigate dynamic protein abundance changes in chicken bursa of Fabricius at 7, 14, 21, and 28 days post-infection (dpi). Key proteins were analyzed by bioinformatics. When these data were combined with histopathological and electron microscopic observations, we gained valuable molecular pathological insights into REV infection in this organ, to generate a scientific theoretical basis for REV prevention and treatment in chickens.

## Materials and Methods

### Animals and the Virus Strain

Fourteen 1-day-old SPF chickens were purchased from Harbin Veterinary Research Institute of China Academy of Agricultural Sciences. REV-T (CVC No. CACCAV107) strains were purchased from the Center for Veterinary Culture Collection (CVCC). REV proliferation in chicken embryo fibroblasts, TCID50, was 10^4.62^/0.1 ml as determined by cell breeding. All animal procedures were approved by the Animal Experiment Ethics Committee of Northeast Agricultural University.

### Experimental Design and Sample Collection

Fifteen 1-day-old SPF chickens were randomly divided into five groups. Non-infected chickens were used as controls (Group C), and chickens at 7, 14, 21, and 28 days after REV infection were assigned in group I7, group I14, group I21, and group I28, respectively. Chickens in the infection groups were intraperitoneally injected with 100 μl of virus suspension and reared separately in isolation rooms. Chickens had *ad libitum* access to food and water. At study end, control and infected chickens were euthanized by CO_2_. The bursa of Fabricius was rapidly excised, washed three times in chilled phosphate-buffered saline (PBS), frozen in liquid nitrogen, and stored at −80°C until required.

### Histopathology

Tissue was fixed in 10% formalin. Based on a previous method (20), tissue was embedded in paraffin, sectioned into 5-μm slices, and stained in hematoxylin and eosin. Morphological changes were observed using light microscopy (Nikon, H600, Japan). Ultra-pathological observations were conducted according to a previous method (20). Briefly, the bursa of Fabricius was fixed in 2.5% glutaraldehyde and 1% osmic acid. Tissue was then dehydrated, soaked and embedded, and sliced into 50-nm sections using an ultra-thin microtome. Then tissues were stained with uranium acetate-lead citrate. Ultra-pathological bursa of Fabricius observations were performed using an electron microscope (Hitachi 7650, Tokyo, Japan).

### Sample Processing and Tandem Mass Tag Labeling

Tissue at −80°C was ground to powder in liquid nitrogen, after which 10% trichloroacetic acid/acetone was added. The sample was then precipitated at −20°C for 2 h. After this, the sample was centrifuged at 4,500 g for 5 min, the supernatant discarded, and the pellet washed in precooled acetone precipitation. After being dried, samples were ultrasonically lysed 1% sodium dodecyl sulfate (SDS) and 1% protease inhibitors and then centrifuged at 12,000 g for 10 min 4°C, and the supernatant was transferred to a new tube. Protein concentration was estimated using the bicinchoninic acid protein assay kit (Elabscience Biotechnology Co., Ltd).

Protein digestion was initiated by vortexing samples in precooled acetone and precipitation at −20°C for 2 h. Samples were centrifuged at 4,500 g for 5 min, the supernatant discarded, and the pellet washed twice in chilled acetone. Tetraethylammonium bromide (TEAB) at a final concentration of 200 mM was added after drying precipitation and ultrasonic dispersion precipitation, trypsin was added, and enzymatic hydrolysis was done overnight. Dithiothreitol (DTT) was added to a final concentration of 5 mM, with samples reduced at 56°C for 30 min. Then, iodine acetamide was added to a final concentration of 11 mM, and samples were incubated in the dark at room temperature for 15 min. The trypsin-hydrolyzed peptides were desalted on a Strata XC18 (Phenomenex) column and vacuum freeze-dried. For analysis, peptides were dissolved in 0.5 M of TEAB and labeled according to TMT kit (Thermo Fisher Scientific, USA) instructions. Briefly, labeling reagent was dissolved in acetonitrile, mixed with sample peptides, and incubated at room temperature for 2 h. Then, labeled peptides were mixed, desalted, and vacuum freeze-dried.

### High-Performance Liquid Chromatography Fractionation and Liquid Chromatography–Tandem Mass Spectrometry Analysis

Peptides were dissolved in liquid mobile phase A and separated on an EASY-nLC 1,000 ultra-high performance liquid system. Mobile phase A contained 0.1% formic acid and 2% acetonitrile. Mobile phase B contained 0.1% formic acid and 90% acetonitrile. Peptides were separated by ultra-performance liquid chromatography (UPLC), injected into nanospray ionization (NSI) ion ionization source, and analyzed by QE plus mass spectrometry. The ion source voltage was 2.2 kV, and peptide parent ions and secondary fragments were detected and analyzed by high-resolution Orbitrap. The scanning primary range was 400–1,500 m/z, and the scanning resolution was 70,000. The secondary scanning range was fixed at a starting point of 100 m/z, and the scanning resolution was 17,500. The data acquisition mode used the data-dependent scanning (DDA) program. The signal threshold was 6.3E4 ions/s, the maximum injection time was 50 ms, and the dynamic exclusion time was 30 s. Three biological repetitions were performed. The mass spectrometry proteomics data have been deposited to the ProteomeXchange Consortium *via* the PRIDE partner repository with the dataset identifier PXD024278.

### Database Searches and Bioinformatics Analysis

Secondary mass spectrometry data were retrieved using Maxquant 1.5.2.8 search parameters. The database was Gallus_gallus_9031_PR_2020072 (24,376 sequences). Gene Ontology (GO) annotations were derived from the UniProt GOA database (http://www.ebi.ac.uk/GOA/). GO enrichment analyses classified proteins according to cellular components, molecular functions, or physiological processes. The Fisher exact-test was used to test differentially expressed proteins in the context of identified proteins. A GO enrichment *p*-value < 0.05 was considered statistically significant. Pathway analyses were performed using the Kyoto Encyclopedia of Genes and Genomes (KEGG) online service tool and the KEGG mapper to match proteins to corresponding pathways. We also performed cluster analysis of protein functional enrichment. Firstly, functional classification information and corresponding enrichment *p*-values were collected, and then the functional classification with significant enrichment (*p*-value < 0.05) in at least one protein group was screened. The filtered *p*-value data matrix was first transformed by logarithm of –log10, and then the transformed data matrix was used to classify each function by Z transform. Finally, data sets were obtained after Z transform is used to do unilateral clustering analysis by hierarchical clustering method. Cluster relationships were visualized using Heatmap.2, a function in R language package gplots.

### Western Blotting

Radioimmunoprecipitation assay (RIPA) lysis buffer (Beyotime Biotechnology Co., Ltd., China) was added to tissue samples, homogenized using tissue homogenizer (Sceintz-48, Ningbo, China) at 120 Hz for 12 s, and centrifuged at 4,000 g for 15 min at 4°C; and supernatants were collected in centrifuge tubes. The bicinchoninic acid protein assay kit (Elabscience Biotechnology Co., Ltd., China) was used for protein quantification. Extracted proteins were subjected to SDS–polyacrylamide gel electrophoresis (SDS-PAGE), transferred to nitrocellulose membranes using the Trans-Blot Turbo Transfer System (BIO-RAD Laboratories, Berkeley, USA), and incubated overnight at 4°C with rabbit anti-HSP70 antibody (Biosynthesis Biotechnology Inc.), rabbit anti-P38 MAPK/MAPK14 (Biosynthesis Biotechnology Inc.), rabbit anti-CD40 (Biosynthesis Biotechnology Inc.), rabbit anti-AKT1 (Biosynthesis Biotechnology Inc.), and rabbit anti-eIF2B2 (Biosynthesis Biotechnology Inc.). Membranes were then washed three times in TBS + Tween20 (TBST) and incubated for 2 h at room temperature with goat anti-rabbit IgG/horseradish peroxidase (HRP) antibody (Biosynthesis Biotechnology Inc.). Finally, membranes were washed three times in TBST, and an enhanced chemiluminescence (ECL) kit (Beyotime Biotechnology, Shanghai, China) was used to visualize protein bands. β-Actin was used as an endogenous reference protein. Protein bands were imaged using a multifunctional imaging analysis system (FluorChem R, ProteinSimple, USA). Band intensity was quantified using ImageJ software (Bethesda, MD, USA).

## Results

### Pathological Changes in the Bursa of Fabricius in Reticuloendotheliosis Virus-Infected Chickens

**Figure 1:**
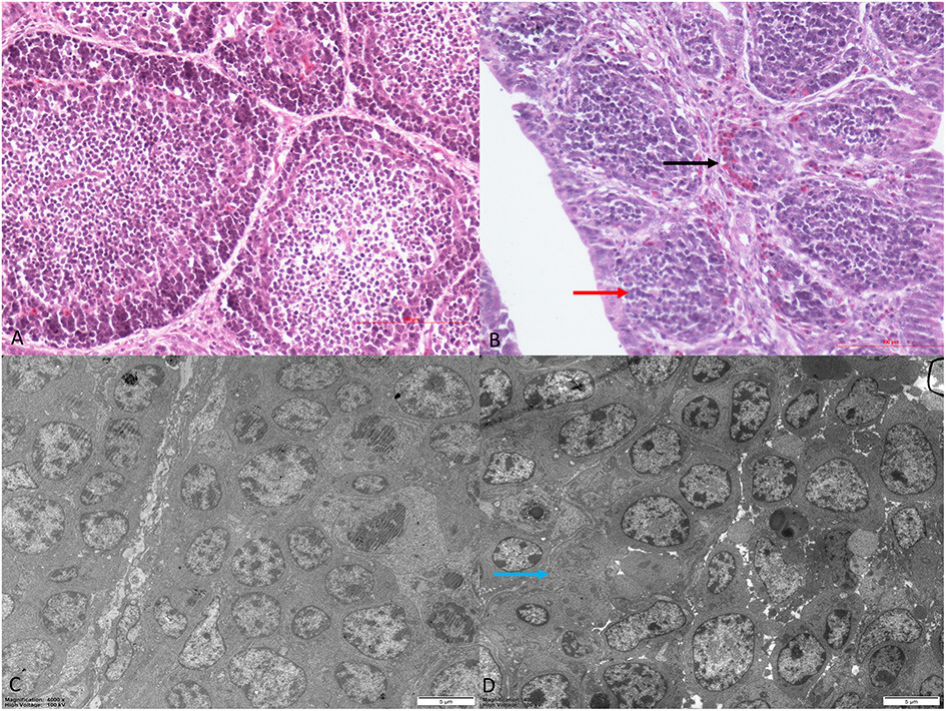
Histological and ultrastructural changes in chicken bursa of Fabricius after reticuloendotheliosis virus (REV) infection. Histological examination of the bursa of Fabricius in the control group **(A)** and REV infection group **(B)** by H&E staining (200×). Histology results showed hyperplasia of the reticular endothelial system (black arrow) and reductions in cortical lymphocytes (red arrow) in the REV infection group. Transmission electron microscopy of the bursa of Fabricius in the control group **(C)** and REV infection group **(D)** (4,000×). Ultrastructural results showed that the REV infection group exhibited apoptosis and necrosis (blue arrow).

Histomorphological and ultrastructural changes in the bursa of Fabricius 21 days after REV infection are shown in Figure 1. Pathological changes in the REV infection groups, when compared with the control group, were manifested as hyperplasia of the reticular endothelial system and reductions in cortical lymphocytes and bursal follicle atrophy. Control group ultrastructure appeared normal under electron microscopy; however, apoptosis, mitochondrial swelling, and necrosis were observed in the REV infection group.

### Tandem Mass Tag-Labeled Proteomics Analysis

Based on TMT labeling and LC-MS/MS approaches, we analyzed bursal protein changes in REV-infected chickens. From MS analysis, 666,226 spectra were obtained, of which 246,403 effective spectra were available. In total, 148,153 peptide segments were identified from spectral analysis, of which specific peptide segments numbered 138,043. In total, 8,193 proteins were identified, of which 7,842 were quantified (Supplementary Table 1). At a *p*-value < 0.05, the variation threshold of significant up-accumulation was set as the variation of differential expression > 1.3, and the variation threshold of significant down-accumulation was set as the variation threshold < 1/1.3.

**Figure 2:**
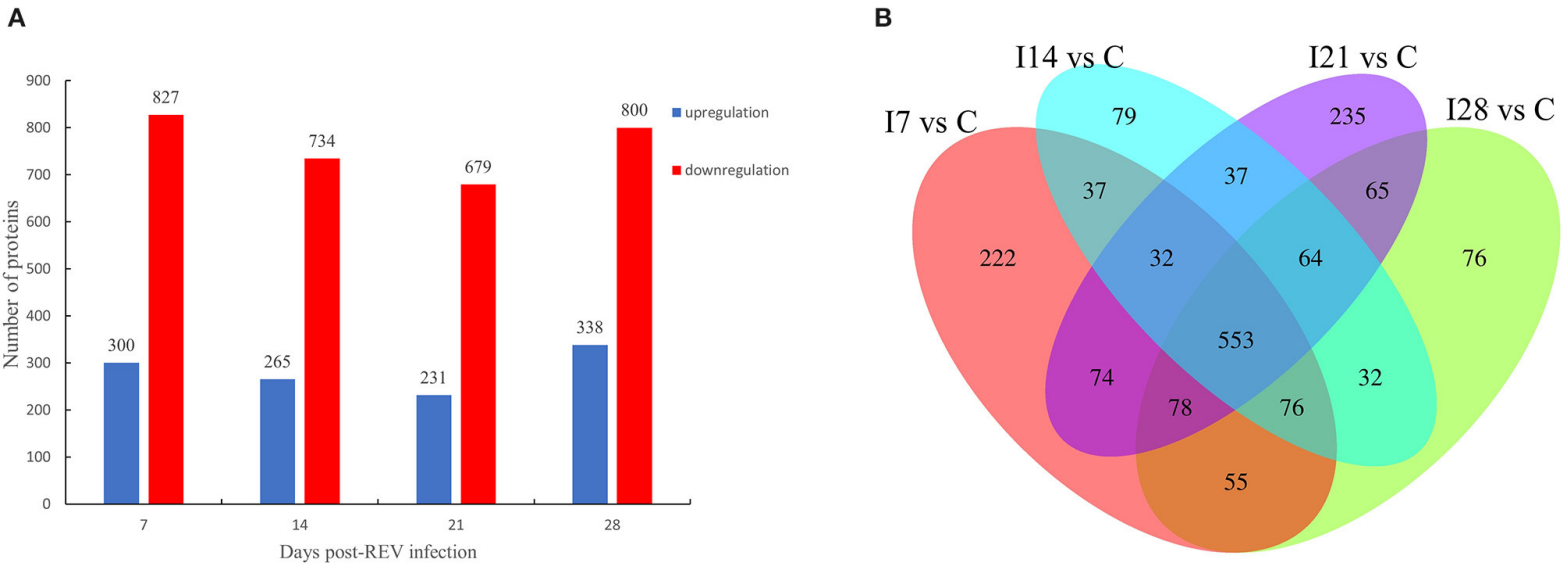
Differentially expressed proteins from chicken bursa of Fabricius infected with reticuloendotheliosis virus (REV). **(A)** Significant and differentially expressed proteins during infection with REV relative to the control group (*p* < 0.05, fold change >1.3 or <1/1.3). **(B)** Venn diagram displays the distribution of differentially expressed proteins during REV infection.

From Figure 2A, our data indicated that 1,127 differentially expressed proteins were identified 7 days after virus infection, including 300 up-accumulated and 827 down-accumulated proteins. At day 14, 999 proteins were identified, including 265 up-accumulated and 734 down-accumulated proteins. At day 21, 910 proteins were identified, including 231 up-accumulated proteins and 679 down-accumulated proteins. At day 28, 1,138 proteins were identified, including 338 up-accumulated and 800 down-accumulated proteins. Based on GO secondary annotations, proteins were divided into 14, 10, and 7 categories based on biological processes, cell composition, and molecular functions, respectively. With the use of KEGG pathway analysis, 52, 48, 43, and 49 pathways were enriched at 7, 14, 21, and 28 dpi, respectively. In Figure 2B, Venn diagram analysis showed that 553 proteins were differentially expressed at four time points after REV infection.

### Functional Classification of Differentially Expressed Proteins

**Figure 3:**
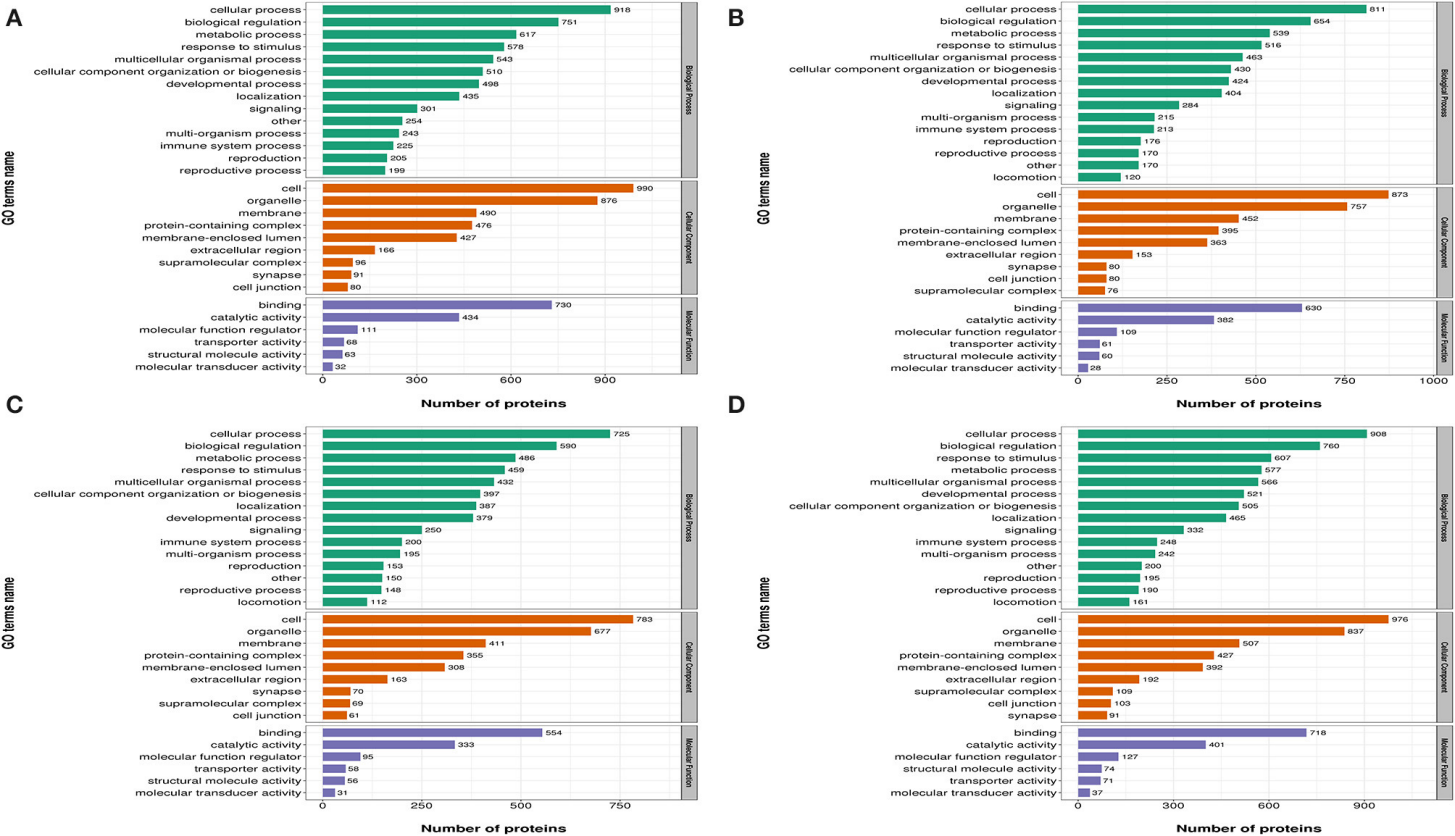
Gene Ontology (GO) analysis of differently expressed proteins based on biological processes, cellular components, and molecular functions. **(A)** 7 dpi. **(B)** 14 dpi. **(C)** 21 dpi. **(D)** 28 dpi.

In Figure 3, differentially expressed proteins, using GO annotations, were classified into three categories: biological processes, cellular components, and molecular functions. Biological process analyses indicated proteins were involved in cellular processes, biological regulation, metabolic processes, responses to stimuli, and multi-cellular organismal processes, over the four time points, post-REV infection. For cell component analyses, proteins were primarily distributed in cell, membrane, and protein-containing complexes at the four time points post-REV infection. These data suggested proteins were involved in a variety of biological processes, cell components, and cell molecular functions.

### Gene Ontology and Kyoto Encyclopedia of Genes and Genomes Cluster Analysis of Differentially Expressed Proteins

**Figure 4:**
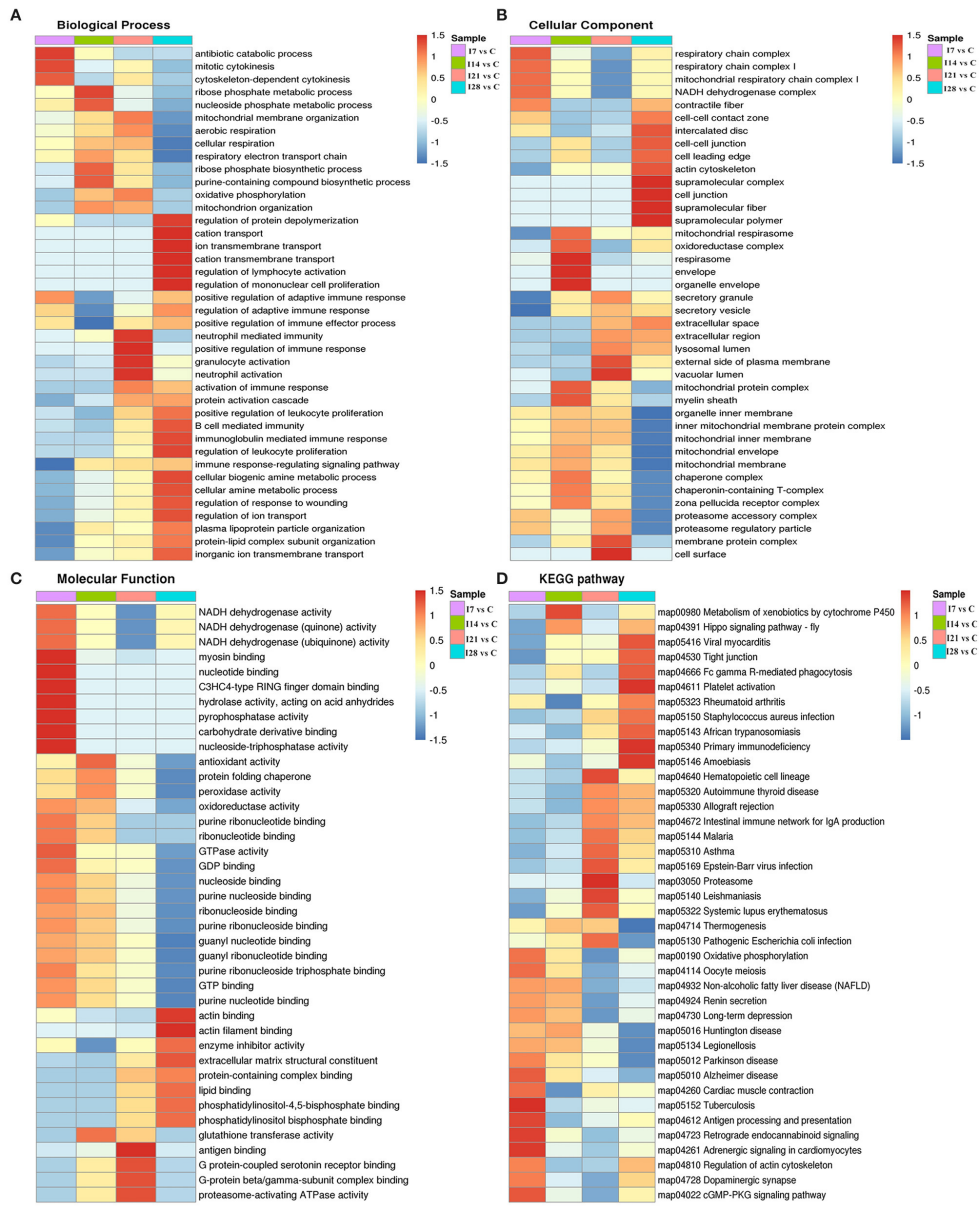
Cluster analysis of differentially expressed proteins based on biological processes, cellular components, molecular functions, and Kyoto Encyclopedia of Genes and Genomes (KEGG) pathways. **(A)** Biological processes. **(B)** Cellular components. **(C)** Molecular functions. **(D)** KEGG pathways.

To further elucidate functional correlations of differentially expressed proteins in our samples, we enriched proteins using GO classifications and KEGG pathway analyses and clustered them. From Figure 4, these analyses showed that for biological processes, on day 7 of infection, proteins were primarily involved in antibiotic catabolic processes, mitotic cytokinesis, and chemokine-dependent cytokinesis. At 14 dpi, proteins were mainly involved in ribose phosphate metabolic and nucleoside phosphate metabolic processes. At 21 dpi, proteins were mainly involved in neutrophil-mediated immunity, positive regulation of immune responses, and granulocyte and neutrophil activation. At 28 dpi, proteins were mainly involved in protein depolymerization regulation, cation transport, ion transmembrane transport, cation transmembrane transport, lymphocyte activation regulation, and mononuclear cell proliferation regulation. Also, proteins showed a high correlation with B cell-mediated immunity, immunoglobulin mediated immune responses and leukocyte proliferation regulation.

For cell component classification, at 7 dpi, proteins were mainly distributed in the respiratory chain and nicotinamide adenine dinucleotide dehydrogenase complexes. At 14 dpi, proteins were mainly distributed in the oxidoreductase complex and organelle envelope. At 21 dpi, proteins were mainly distributed in the vacuolar lumen, the external side of the plasma membrane, membrane protein complexes, and the cell surface. At 28 dpi, proteins were mainly distributed in super-fraction complexes, cell–cell junctions, and the actin cytoskeleton.

For molecular functions, at 7 dpi, proteins were mainly concentrated in phosphatase–triphosphatase activities, nucleotide binding, and myosin binding. At 14 dpi, proteins were mainly concentrated in antioxidant activities, peroxidase activities, and glutathione transferase activities. At 21 dpi, proteins were mainly concentrated in antigen binding, G protein-coupled serotonin receptor binding, and proteasome-activating ATPase activities. At 28 dpi, proteins were mainly concentrated in actin binding, enzyme activity, and extracellular matrix structural inhibitor.

KEGG pathway analysis showed that proteins at 7 dpi were mainly related to antigen processing and presentation, actin cytoskeleton regulation, and the cyclic guanosine monophosphate (cGMP)-dependent protein kinase (PKG) signaling pathway. At 14 dpi, proteins were related to xenobiotic metabolisms *via* the cytochrome P450 signaling pathway. At 21 dpi, proteins were mainly related to the immune response signaling pathway caused by exogenous pathogenic microorganisms such as Epstein–Barr virus infection. At 28 dpi, proteins were mainly associated with Fc γR-mediated phagocytosis and primary immunodeficiency signaling pathways.

### Cluster Analysis of Protein Abundance Patterns

**Figure 5:**
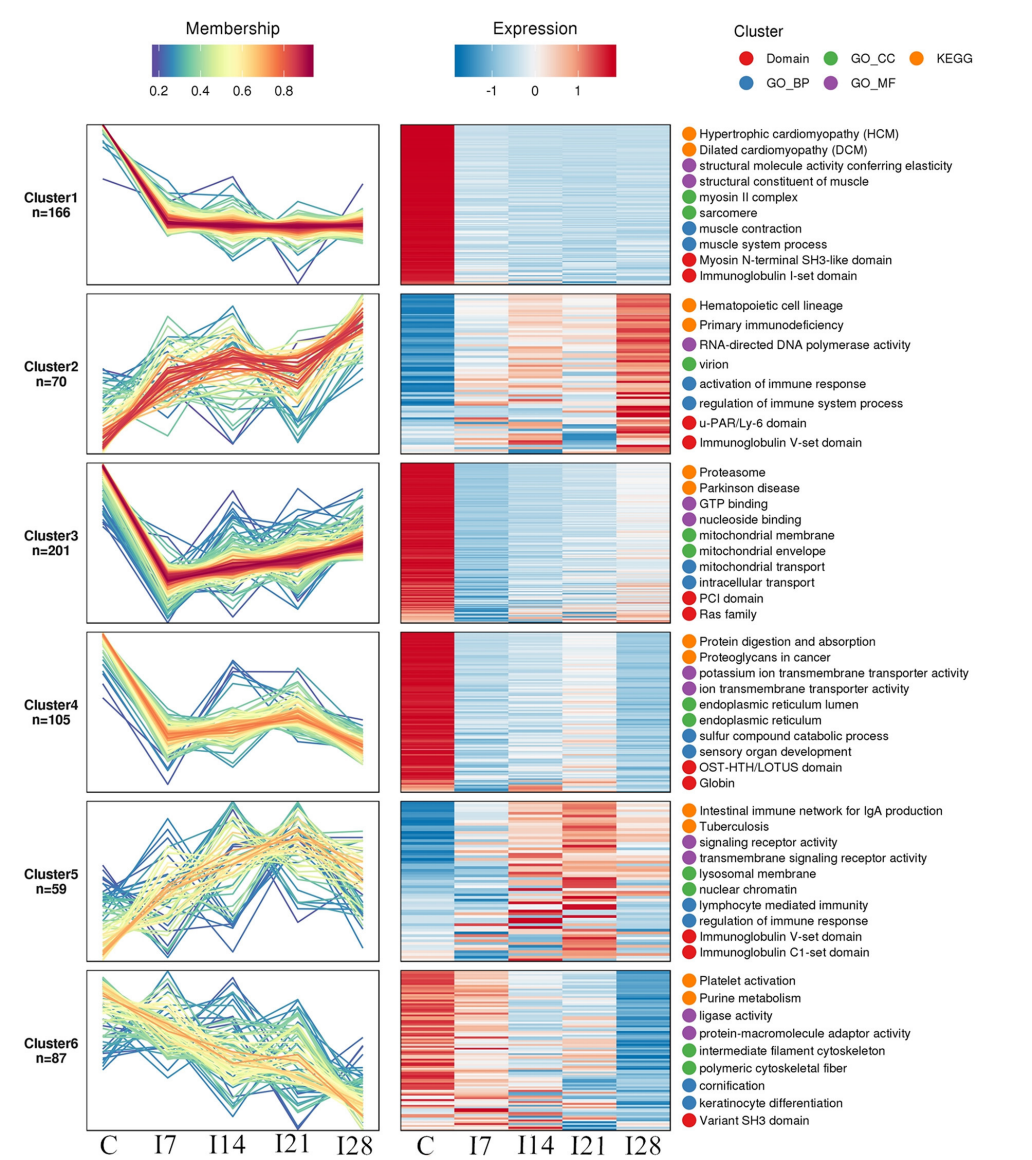
Functional clustering of differentially expressed proteins. All time profiles were clustered by a fuzzy clustering algorithm to determine modules of co-regulated proteins. The first two most significantly enriched entries in the Gene Ontology (GO)/Kyoto Encyclopedia of Genes and Genomes (KEGG)/Domain enrichment analysis are displayed on the right side of the corresponding cluster expression pattern clustering graph.

We identified 8,193 proteins using proteomics quantitative analyses (Supplementary Table 2). To screen for significant changes in protein abundance relative protein expression was converted to Log2 logarithm, and proteins with SD > 0.4 were screened. The remaining 688 proteins were used for Mufzz expression pattern clustering analysis. Analysis parameters included the following: cluster number “k” was 6, and clustering ambiguity “m” was 2. Data are shown in Supplementary Table 3. The cluster column reflects corresponding protein abundance pattern class (cluster), and the same cluster protein has a similar expression transformation trend. Proteins were divided into six classes using Mfuzz. To study the biological characteristics of proteins in different classifications, we performed enrichment analyses of proteins in different classifications in GO/KEGG/Domains (Supplementary Figures 1–5). To facilitate the display of all the analysis results, the first two most significantly enriched entries in the GO/KEGG/Domain enrichment analysis results are displayed on the right side of the corresponding cluster expression pattern clustering graph (Figure 5).

Our Mfuzz analyses indicated that proteins in cluster 1, including biological processes and molecular functions of movement of cell or subcellular components, actin cytoskeleton organization, supramolecular complex, cytoskeleton, structural molecule activity, and the immunoglobulin I-set domain, were decreased at day 7 after REV infection, and stable trends were observed at 7–28 days. Proteins in cluster 2, including intestinal immune networks for IgA production, calcium signaling pathways, NF-κB signaling pathways, adaptive immune responses, and lymphocyte-mediated immunity, exhibited increased expression after infection. Proteins in cluster 3, including intracellular protein transport, mitochondrial membrane organization, G protein-coupled receptor binding, and GTPase activity, were decreased within 7 days of REV infection but showed a steady increase from 7 to 21 days. In cluster 4, related biological processes and protein domains appeared in protein digestion and absorption, endoplasmic reticulum, globin, and Tudor domains. Protein expression decreased and then increased slowly at 21 dpi. Proteins in cluster 5, including antigen processing and presentation, intestinal immune networks for IgA production pathways and adaptive immune responses, lymphocyte-mediated immune biological processes, immunoglobulin receptor activity, and transmembrane signaling receptor activity, were increased within 21 dpi, with a slight decrease at 21–28 days. Proteins in cluster 6, including purine metabolism, platelet activation, ligase activity, intermediate filament cytoskeleton pathways, or molecular functions, showed a continuous decrease in expression within 28 dpi.

### Validation of Mass Spectrometry Data Using a Western Blotting Strategy

**Figure 6:**
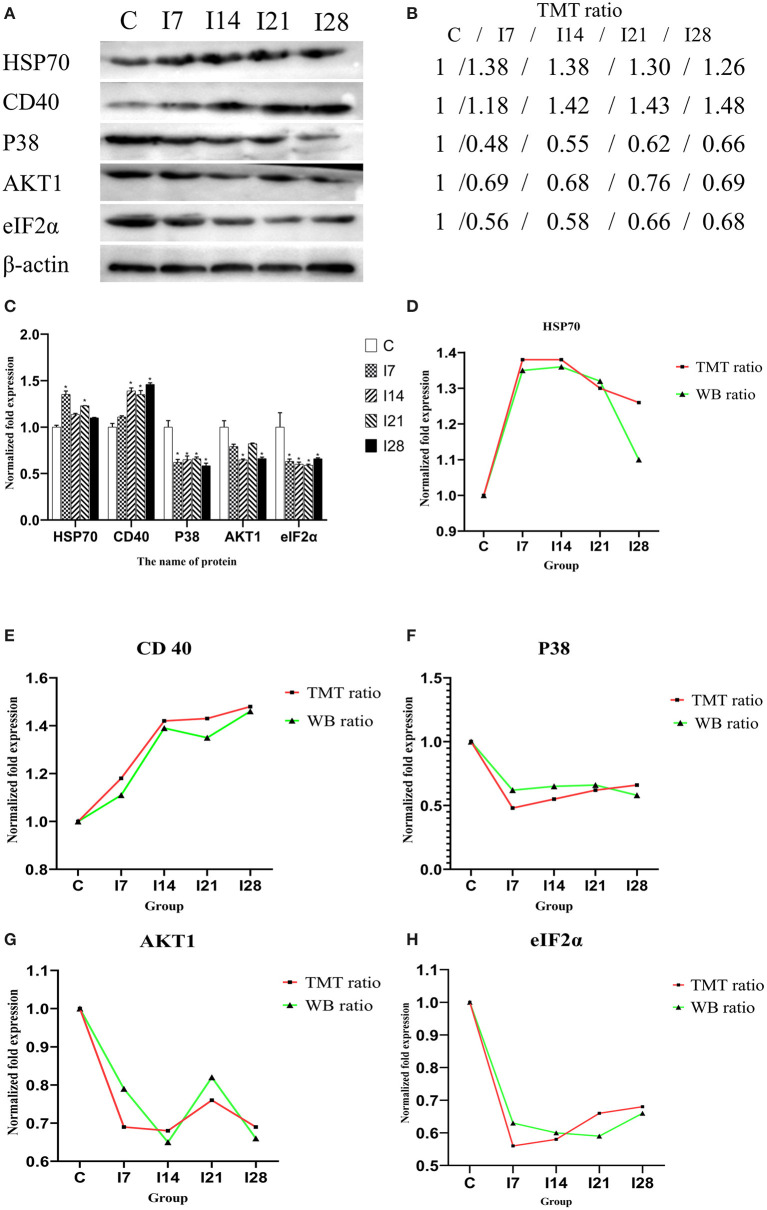
Verification of differentially expressed proteins by Western blotting. **(A)** Representative Western blotting images of HSP70, CD40, p38, AKT1, and eIF2α. **(B)** Tandem mass tag (TMT) ratios (C/I7/I14/I21/I28). **(C)** Western blotting quantitative analysis on the change of protein expression of HSP70, CD40, p38, AKT1, and eIF2α. **(D–H)** The FC ratios between TMT and Western blotting (WB) data of HSP70, CD40, p38, AKT1, and eIF2α.The expression trends of proteins identified by WB analysis-matched proteomics data. *P < 0.05 indicates a significant difference compared with the control group.

To verify MS data accuracy, we randomly selected five proteins to investigate expression by western blotting (Figure 6). The results showed that HSP70, p38, and CD40 were up-accumulated, and AKT1 and eIF2α down-accumulated consistently with MS data.

## Discussion

The innate immune system recognizes pathogen-associated molecular patterns through the pattern recognition receptor (PRR), which is the first line of defense against pathogens. PRRs include toll-like receptors (TLRs), retinoic acid-inducible gene 1 (RIG-1)-like receptors (RLRs), nucleotide-binding oligomerization domain (NOD)-Leucin Rich Repeats (LRR)-containing receptors (NLRs), and C-type lectin receptors (CLRs). After the corresponding viral RNA or DNA was identified, signal transduction is initiated, and interferon (IFN) is expressed to induce antiviral effects (21). Both TLR7 and TLR21 in poultry activate IFN through the MyD88 pathway to target virus invasion (22, 23). Previous studies have shown that after MDV infection in chickens, TLR7, MyD88, and IFN-I expression were decreased, indicating that MDV infection inhibited immune responses (24). Our data suggested differentially expressed proteins after REV infection were involved in PRR-related signaling pathways. MyD88 expression was down-accumulated at the four time points after infection, suggesting that IFN expression may also be affected.

NF-κB plays key roles in mediating cell inflammatory and immune responses. Several NF-κB pathway activation factors are known, including tumor necrosis factor (TNF)-α, interleukin (IL)-1β, IL-2, IL-6, IL-8, IL-12, inducible nitric oxide synthase (iNOS), cyclooxygenase-2 (COX-2), chemokines, adhesion molecules, and colony-stimulating factors (14). In this study, nuclear factor NF-κB p100 and NF-κB inhibitor epsilon are NF-κB inhibitory proteins. Their expression increased at 7–14 and 21–28 days after infection, suggesting that REV infection reduced NF-κB expression, thereby inhibiting immune responses.

IL-18 was also up-accumulated at the four time points after REV infection. IL-18 is as a pro-inflammatory cytokine and a member of the IL-1 cytokine family, which induces IFN-γ expression. It also exhibits multidirectional effects during inflammation (25). Previous studies have reported that tripartite motif-containing protein 39 (TRIM39), as a protein encoded by IFN-stimulated genes (ISGs), exhibits antiviral activity (26). ISG expression depends on IFN induction. In this study, TRIM39 was up-accumulated at four time points after virus infection; thus, TRIM39 expression may be induced by IFN. In our cluster analysis of protein expression patterns, we found that immune-related pathways, including the intestinal immune network for IgA production, calcium signaling pathways, and NF-κB signaling pathways, were activated at four time points after viral infection, suggesting REV infection activated immune responses to resist viral invasion. Previous studies have shown that during REV infection, IL, IFN, and TNF showed significant regulation, suggesting that REV infection disordered the cytokine network, affecting immune functions (14). Therefore, the differentially expressed proteins MyD88, NF-κB p100, NF-κB inhibitor epsilon, TRIM39, and IL-18 may be associated with the innate immune system of chickens infected with REV. Future studies are required to confirm these data.

Viral infection affects the cell cycle and improves viral replication efficiency by disrupting host cell functions, such as RNA virus assembly for longer time by blocking cell cycle, thereby improving viral genome replication (27). Infectious bronchitis virus inhibits cell division and arrests cells in S/G2 phase, affecting cell growth (28, 29). Phosphatidylinositol-4,5-bisphosphate 3-kinase (PI3K)/protein kinase B (AKT) promotes cell proliferation and inhibits cell apoptosis. MDV activates the PI3K/AKT pathway *via* interactions between viral protein Meq and PI3K p85 subunit, to delay cell apoptosis and promote virus replication (30). Some oncogenic viruses also promote host cell proliferation, e.g., Epstein–Barr virus EB nuclear antigen 3C promotes cell proliferation by regulating cyclin D2 and inhibiting the apoptosis of uncontrolled proliferation (31, 32). It was previously reported that Newcastle disease virus (NDV) replication in cells leads to G0/G1 phase arrest in infected cells, permitting NDV proliferation. Cyclin D1 expression was also significantly decreased after NDV infection. Similarly, during NDV infection, the PERK-EIF2α-ATF4-CHOP signaling pathway was also implicated in G0/G1 cell cycle arrest (33). In our study, CDK5, PDK-1, PDK-3, and AKT1 were down-accumulated at 7, 14, 21, and 28 days after REV infection. Importantly, KEGG pathway analysis and expression pattern clustering analysis confirmed this observation. We speculate this may be due to the promotion of viral replication by extending the cell cycle after viral infection, but this requires further investigation.

During oxidative stress, highly active molecules such as reactive oxygen species (ROS) are produced; and when antioxidant clearance is exceeded, abnormal gene expression, receptor activity, aberrant cell proliferation, or cell death ensues (34). After infection, the body is generally in a chronic non-acute oxidative stress state. Tissue damage caused by oxidative stress is not only a pathological consequence of viral infection but also a key pathogenic mechanism (35). Peroxiredoxin (PRDX) is a vital peroxidase reductase that synergistically acts with thioredoxin to effectively remove peroxides metabolism (36, 37). Catalase (CAT) is an efficient enzyme that scavenges and decomposes hydrogen peroxide to protect cells from oxidative damage (38, 39). The main function of glutathione peroxidase (GPX) is to catalyze glutathione to oxidized glutathione, thereby reducing lipid hydroperoxides to hydroxyl compounds and blocking lipid peroxidation (40). In our study, PRDX4, PRDX6, and GPX3 expression were down-accumulated at 7 and 14 days after REV infection, while CAT and peroxidasin (PXDN) expression were up-accumulated at 7–21 days. In addition, SOD3 was up-accumulated at 14–28 days. We speculated that oxidative stress persisted in the early and middle stages of REV infection and was alleviated in later stage. Our cluster analysis showed that differentially expressed proteins were enriched for oxidoreductase and antioxidant activity after viral infection. Histopathological and ultrastructural changes of the bursa of Fabricius showed that after REV infection, lymphocyte numbers decreased, inflammatory cell infiltration increased, the mitochondria swelled, the nuclear membrane dissolved, and nucleoli levels increased. These results may have been due to decreased localized antioxidant capacity, potentially accumulating oxidative molecules and resulting in immune cell damage at the bursa of Fabricius. Our previous chicken studies also indicated that ROS, reactive nitrogen species (RNS), and malondialdehyde (MDA) levels in the thymus increased, and GPX, CAT, superoxide dismutase (SOD) levels decreased significantly after REV infection, indicating oxidation levels increased and antioxidant capacity decreased, resulting in oxidative stress (20). This was consistent with our analysis in the bursa of Fabricius. In addition, previous studies showed that NDV caused oxidative stress in chickens. Adding vitamin E to diets significantly reduced intestinal oxidative stress levels and reduced the impact of the virus on the chickens (41, 42). Sargassum polysaccharides also resisted IBDV infection by improving the antioxidant capacity and cytokine levels of chicken bursal cells (43). This suggested that some poultry viruses can cause oxidative stress, and antioxidant therapy may have significant therapeutic effects toward viral infection (44). We speculate that adding antioxidants to diets could prevent and treat REV in the future, but this requires further investigation.

Heat shock proteins (HSPs) are highly conserved functionally related proteins, widely expressed in all cells. They are divided into different families based on molecular weight and include HSP100, HSP90, HSP70, HSP60, HSP40, and small HSP (15–40 kDa) (45–48). When cells are stimulated by physical and chemical environments, i.e., virus infection, temperature changes, and starvation, HSPs are rapidly expressed to stabilize protein folding (49). HSP90 plays an important role in viral entry into cells. In previous studies, upon cell treatment with HSP90 inhibitors, enterovirus A71, Japanese encephalitis virus, and dengue virus access to cells was variably blocked (50–54). During Zika virus infection, viral entry and RNA replication are regulated by glucose-regulated protein 78 (GRP78) (55, 56). The HSP40 family protein, Dna J HSP family (Hsp40) member 14 (DNAJC14), is involved in flavivirus viral replicase complex synthesis (57). Similarly, DNAJC14 deletion affects herpes complex virus replication (58). In our study, the expression levels of HSPH1 and DnaJ HSP family (Hsp40) member A4 (DNAJA4) were up-accumulated at 7, 14, 21, and 28 days after REV infection. In addition, heat shock cognate 71-kDa protein (HSPA8) and HSP family A (Hsp70) member 4 like (HSPA4L) were only up-regulated at 7 and 14 days after REV infection. Therefore, we speculated that REV might promote viral replication by up-accumulated HSPH1, DNAJA4, HSPA8, and HSPA4L, especially at 14 days after REV infection. These results show that HSPs play important roles during REV infection. In the future, we will study the relationship between HSP family members and REV virus replication.

In summary, we used TMT-labeled quantitative proteomics to study dynamic changes in chicken bursal proteins at 7, 14, 21, and 28 dpi (compared with protein abundance before infection). GO, cluster, enrichment KEGG pathway, and pattern expression cluster analyses identified differentially expressed proteins and corresponding biological processes related to REV infection in chickens. This is the first time quantitative proteomics have been used to study protein changes in chicken bursa of Fabricius infected with REV. In future studies, we will focus on antioxidant treatments after REV infection and investigate relationships between REV infection, the HSP family, and endoplasmic reticulum stress.

## Data Availability Statement

The datasets presented in this study can be found in online repositories. The names of the repository/repositories and accession number(s) can be found below: http://www.proteomexchange.org/, PXD024278.

## Ethics Statement

The animal study was reviewed and approved by Animal Experiment Ethics Committee of Northeast Agricultural University.

## Author Contributions

SZhe designed the experiments and revised manuscript. DY analyzed the data and drafted manuscript. XL performed the experiments of bioinformatics analysis. SZha performed the experiments of morphological analysis. All authors contributed to the article and approved the submitted version.

## Conflict of Interest

The authors declare that the research was conducted in the absence of any commercial or financial relationships that could be construed as a potential conflict of interest.
