# Supplementary figures and images for: Tandem Mass Tag-Based Quantitative Proteomic Analysis of Chicken Bursa of Fabricius Infected With Reticuloendotheliosis Virus
Source: Front Vet Sci. 2021 May 25;8:666512. doi: 10.3389/fvets.2021.666512 (PMC8186552; doi:10.3389/fvets.2021.666512)

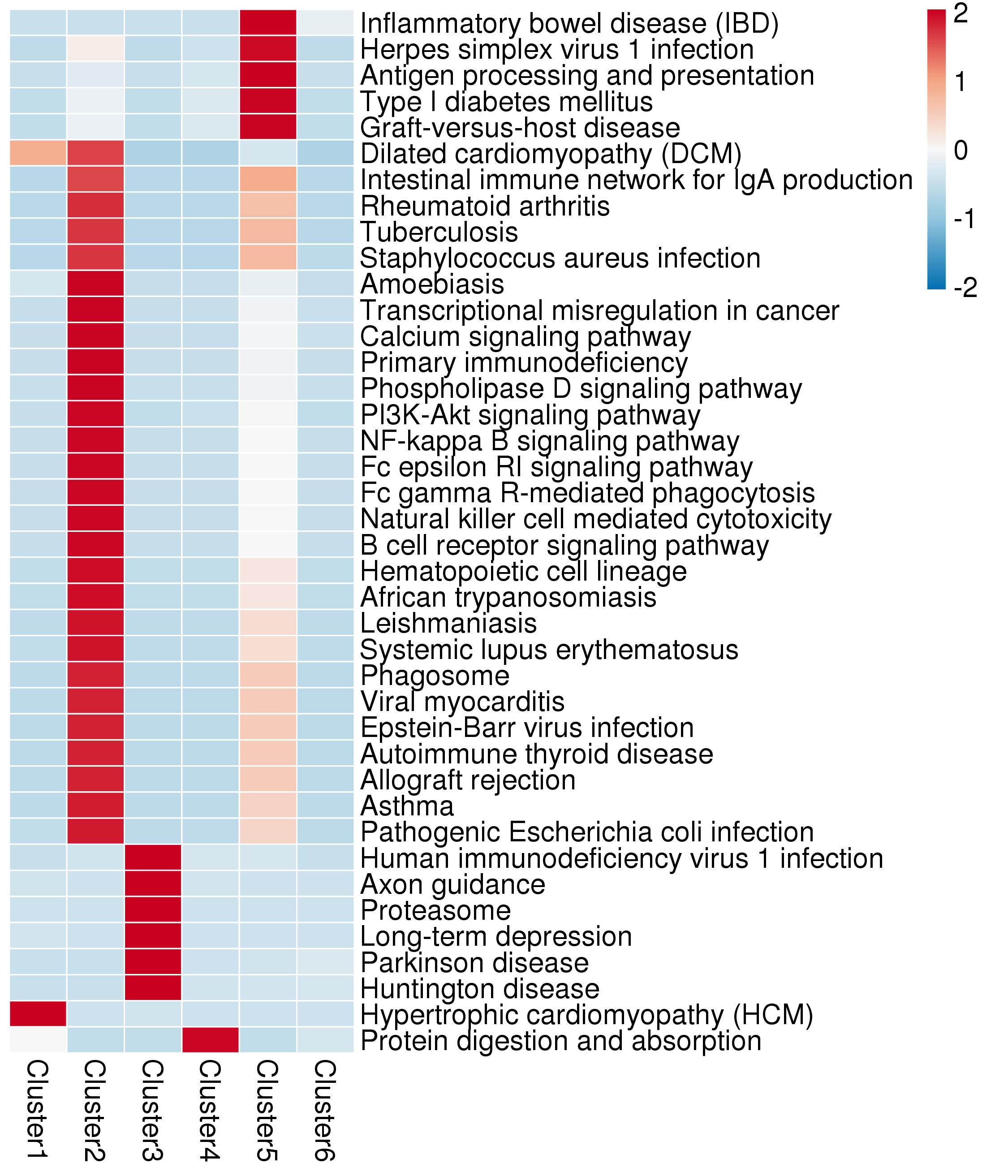

Supplement: Supplementary file 1 [file Image_1.PNG]

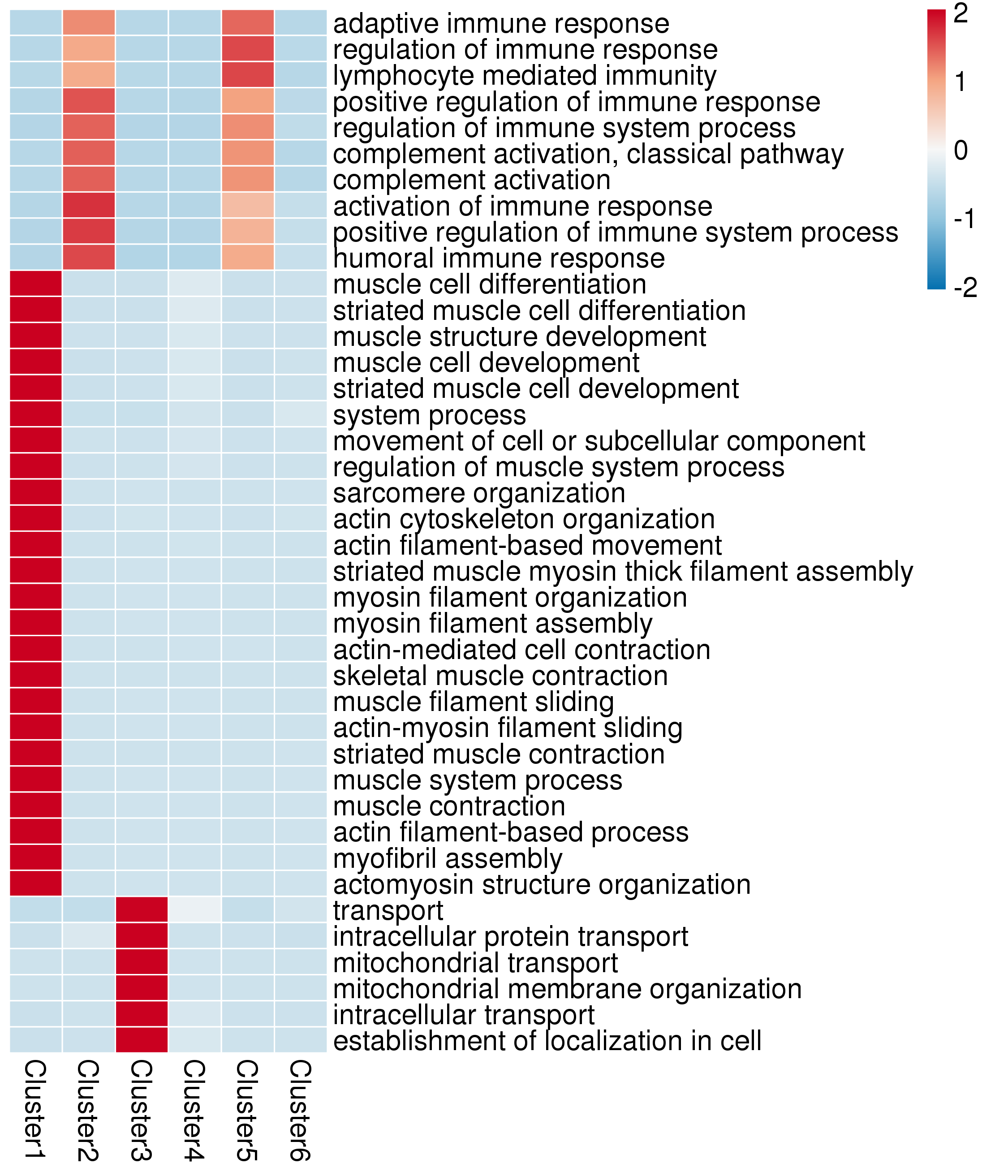

Supplement: Supplementary file 2 [file Image_2.PNG]

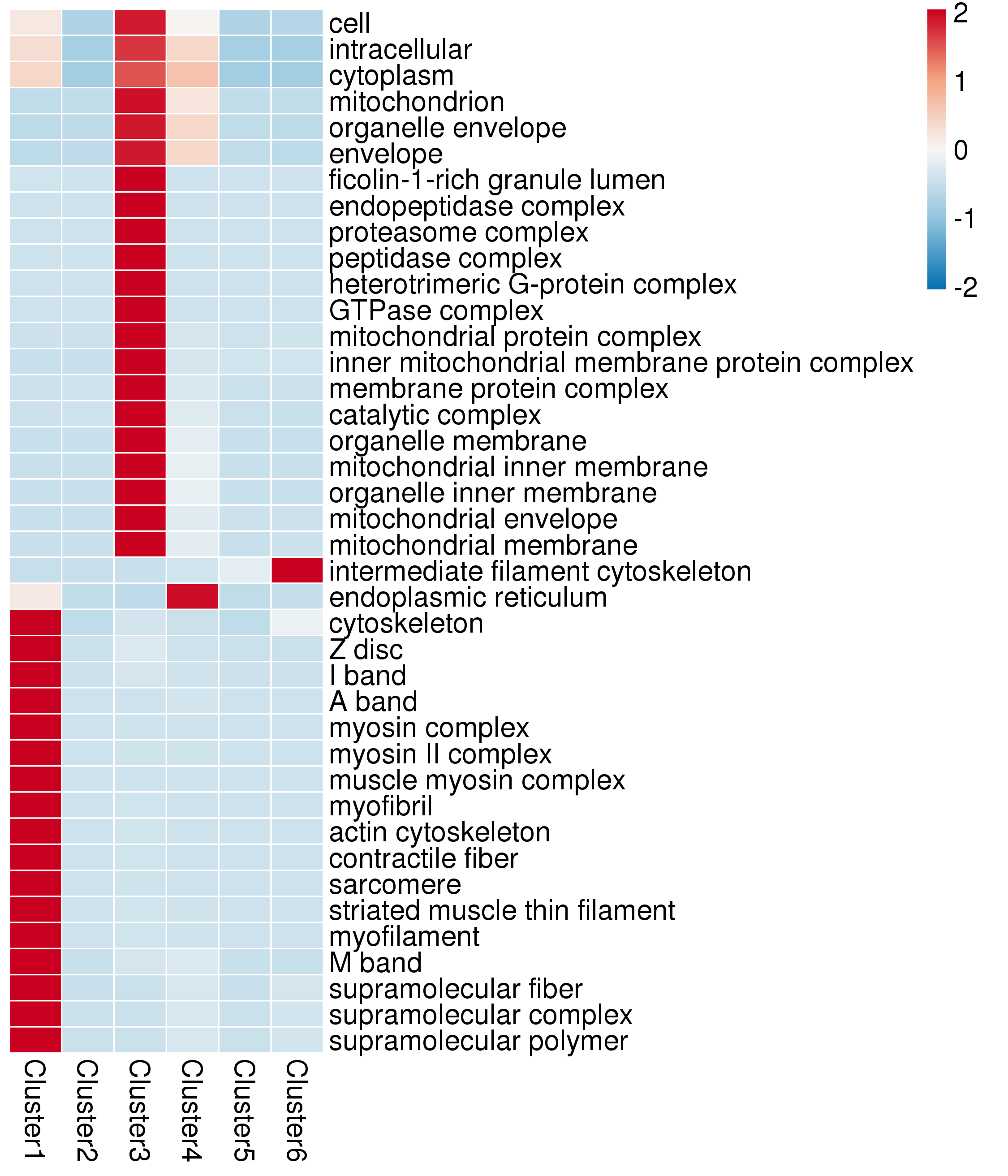

Supplement: Supplementary file 3 [file Image_3.PNG]

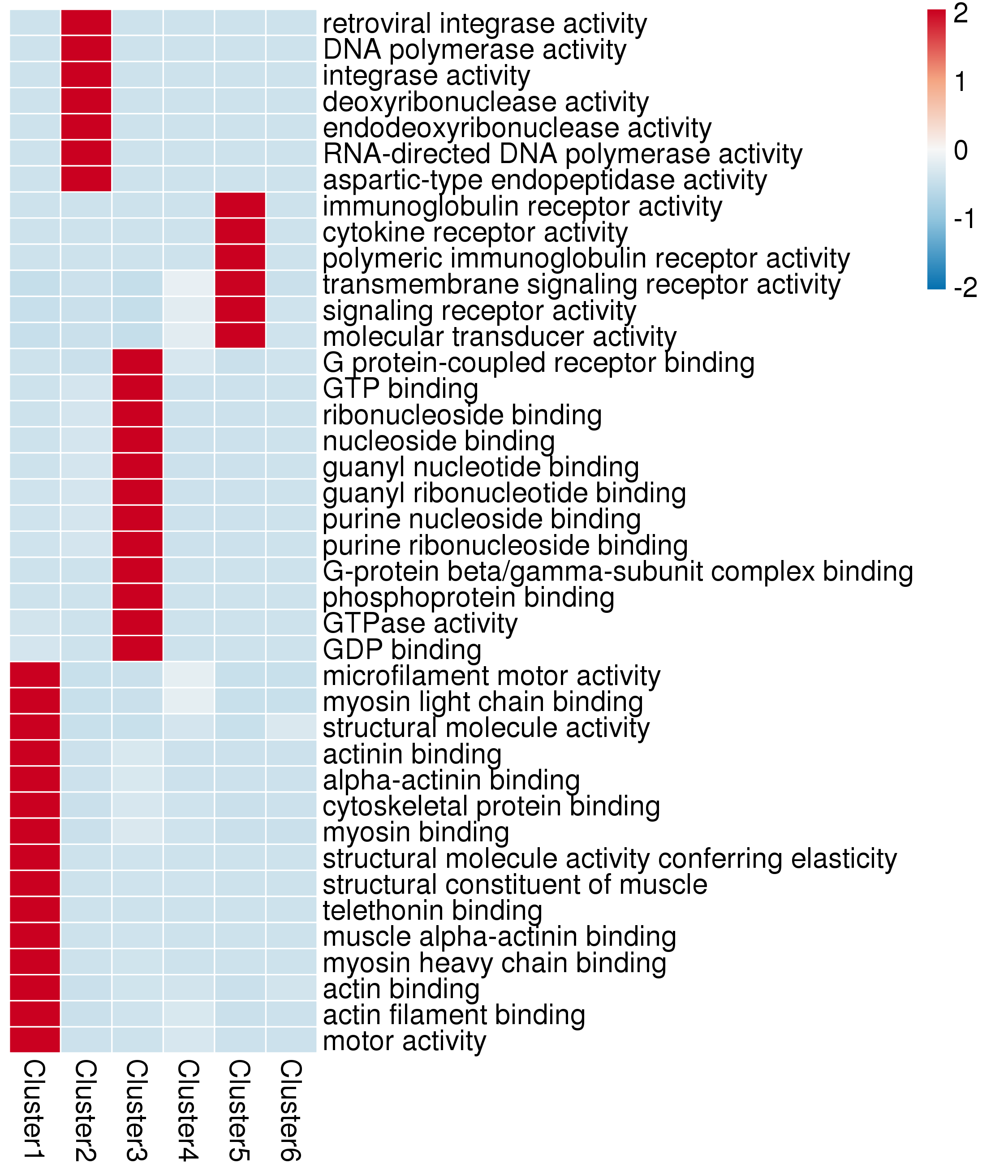

Supplement: Supplementary file 4 [file Image_4.PNG]

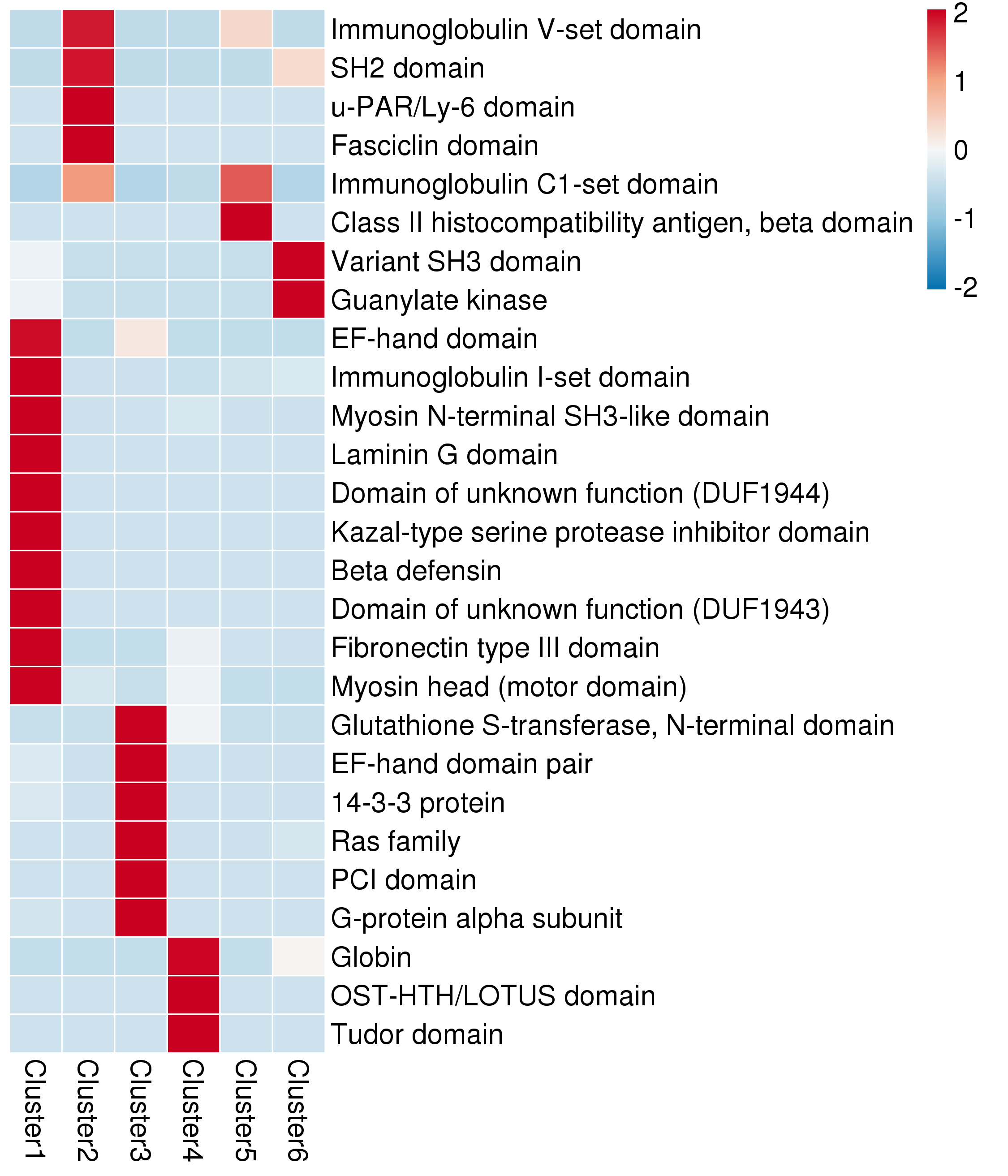

Supplement: Supplementary file 5 [file Image_5.PNG]
